# A Custom Solution for Acoustic Startle Response Setup with Spike2-Based Data Acquisition Interface

**DOI:** 10.3390/mps6030057

**Published:** 2023-06-08

**Authors:** Arseniy Pelevin, Natalia Kurzina, Vladislav Zavialov, Anna Volnova

**Affiliations:** 1 Faculty of Biology, St Petersburg University, 199034 Saint Petersburg, Russia; 2 Institute of Translational Biomedicine, St Petersburg University, 199034 Saint Petersburg, Russia

**Keywords:** acoustic startle response (ASR), prepulse inhibition (PPI), attention, Spike2 script

## Abstract

This article presents a low-cost and flexible software solution for acoustic startle response (ASR) test that can be used with a Spike2-based interface. ASR is a reflexive response to an unexpected, loud acoustic stimulus, and prepulse inhibition (PPI) is a phenomenon in which the startle response is reduced when preceded by a weak prestimulus of the same modality. Measuring PPI is important because changes in PPI have been observed in patients with various psychiatric and neurological disorders. Commercial ASR testing systems are expensive, and their closed source code affects their transparency and result reproducibility. The proposed software is easy to install and use. The Spike2 script is customizable and supports a wide range of PPI protocols. As an example of PPI recording, the article presents data obtained in female rats, both wild-type (WT) and dopamine transporter knockout (DAT-KO), showing the same tendency as the data obtained in males, with ASR on a single pulse higher than ASR on prepulse+pulse, and PPI reduced in DAT-KO rats compared to WT.

## 1. Introduction

Prepulse inhibition (PPI) refers to the phenomenon in which an animal or human’s startle response to a strong stimulus is reduced when preceded by a weak prestimulus of the same modality [1,2,3]. The degree of this reduction (PPI) is calculated as a ratio of the response intensity to the paired weak prestimulus and strong stimulus, to the intensity of the response to a single strong stimulus [3,4,5]. The PPI phenomenon is based on the sensorimotor gating mechanism and involves brainstem structures as well as some diencephalon and the basal ganglia areas [3,6]. It is clinically significant due to the observed PPI changes in patients with schizophrenia, schizotypal personality disorder, obsessive-compulsive disorder, Huntington’s disease, and other psychiatric and neurological disorders [7]. PPI is commonly performed in animal models of human disease to investigate mechanisms of impairment and drug testing. [4,8,9,10]. Various types of startle stimuli are applied, including acoustic, visual, and tactile [3,4].

Commercial acoustic startle response (ASR) and PPI testing systems are designed for both rats and mice. These systems present experimental animals with startle stimuli, detect startle responses, and provide sound isolation. Examples of these commercial systems include SR-LAB (San Diego Instruments, San Diego, CA, USA), MED-ASR-PRO1 (Med Associates, Fairfax, VT, USA), StartFear (Panlab, Barcelona, Spain), Startle Reflex apparatus (Imetronic, Marcheprime, France), and Startle Reflex system (Kinder Scientific, Chula Vista, CA, USA). The provided software for these setups allows the experimenter to define the number of stimuli of each of the three types (weak prepulse, strong pulse, and paired prepulse+pulse stimulus), their volume, and the time between stimuli. After all the parameters are set, the system executes the program automatically without any further experimenter’s participation. Although commercial systems provide all the necessary features for the PPI procedure, their high cost might be a barrier in using them. Furthermore, it is not always possible to buy expensive equipment for just one series of experiments. Another downside of the commercial systems is the closed source code, which affects their transparency and result reproducibility, thus making it impossible to upgrade or repurpose the setups [11].

Several scientific groups have already developed custom systems for ASR and PPI protocols. Gerum et al. [12] suggested a Python-based setup for PPI and gap-prepulse inhibition of the acoustic startle reflex (GPIAS) protocols [13], which requires an additional soundcard for sound presentation and a data acquisition card for recording and digitization of analog signal from acceleration sensor. A similar solution realized with a graphical programming language G in the integrated design environment for data collection and processing (LabView) was proposed by Pevtsov et al. [14]. Virag et al. [11] repurposed a kitchen scale for startle response measurement. In this open-source solution, the hardware is controlled by Arduino boards, while the graphical user interface for PC is written in Python, and data analysis is performed using R. A possible further step would be to implement the PPI protocol on hardware already present in laboratories, familiar to researchers, and allowing simultaneous conduction of electrophysiological experiments.

CED Power1401, produced by Cambridge Electronic Design Ltd. in Cambridge, UK, is a data acquisition and control interface widely used in neuroscience research. The interface operates with Spike2 software (versions 4–10, Cambridge Electronic Design, Cambridge, UK) and allows installing custom scripts in Spike2 scripting language. Electrophysiological registration can also be performed in conjunction with startle response tests. High precision and accuracy of the CED Power1401 make it suitable for experimental protocols that require high temporal resolution. Furthermore, it allows both analog–digital input and digital–analog output, making it ideal for the PPI procedure. The PPI setup does not require additional complex equipment if CED interface with Spike2 software is already available in the laboratory and can be assembled by researchers themselves.

CED Power1401 allows one to control almost any analog device based on the Spike2 software. While there is a Spike2 script available for generating paired pulses and analyzing results (PPulse.s2s, published on the official CED website [15]), it lacks some of the parameters typically required for the PPI protocol, such as sound and white noise generation, sound volume settings, and presentation order of different types of stimuli. In our work, we tried to fill this gap. The CED Power1401 and Spike2 have been used for stimulus presentation in the PPI procedure previously [2], but no software or setup description has been published.

Setting the parameters of the acoustic signals to align with the research objectives is the primary challenge in implementing the PPI paradigm. The PPI paradigm requires an adjustment of various parameters and specifications to fit the experimental design [4]:

1. Length and amplitude of acoustic pulses;
2. Intervals between prestimulus and stimulus in paired pulses (interstimulus interval, ISI);
3. Intervals between consecutive stimuli presentations (intertrial interval, ITI);
4. Variability of presentation frequency to prevent the development of reflexes for the next stimulus;
5. Number of stimuli of each type and their presentation order;
6. Synchronization of ASR amplitude registration and the timing of stimuli for further analysis;
7. Calibration of output voltage to the sound volume of the existing equipment.

This article proposes a flexible solution for acoustic startle response tests that meets all of the aforementioned requirements. It is simple to set up and operate and can be cost-effective for a laboratory that already has CED Power1401 or a similar Spike2-based interface installed.

The software has been successfully used in our previous studies, and the results have been published earlier [9,10]. The current paper provides an example of using Spike2-based data acquisition interface to assess PPI in wild-type and knockout rats.

## 2. Materials and Methods

### 2.1. Description of the Script Interface and System Components

**Figure 1:**
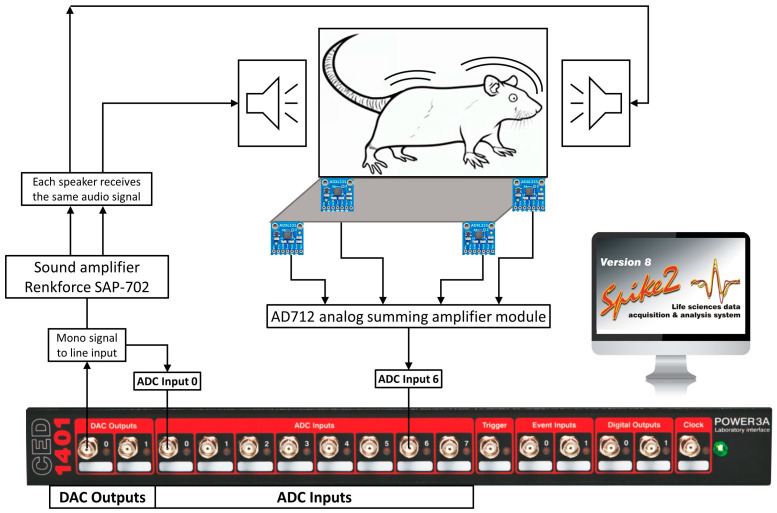
The block diagram of the ASR system components. Signal presentation and response recording is performed with a CED Power1401-3A data acquisition and control interface controlled with Spike2 8.08 software. Sound amplifier receives mono signal from the DAC output 0 channel and sends the same audio signal to both loudspeakers. The controlling signal is also sent to an ADC input for time synchronization. Four accelerometers mounted in the platform provide analog signal to the AD712 analog summing amplifier, which sends the summed signal to another ADC input.

The procedure requires several pieces of equipment, including a data acquisition and control interface CED Power1401, controlled by Spike2 8.08 software (CED Power1401-3A, Cambridge Electronic Design, Cambridge, UK), a sound amplifier (Renkforce SAP-702, Conrad Electronic SE, Hirschau, Germany), a cage with two loudspeakers installed in the wall to present acoustic stimuli (Speaka Special DL-1152BK4, Conrad Electronic SE, Hirschau, Germany), a platform with four vibration sensors (ADXL335 accelerometer, Adafruit Industries, New York, NY, USA) to capture the animal’s startle response, and an analog summing amplifier module with AD712 operational amplifiers (Analog Devices, Wilmington, MA, USA) that combines inputs from the four accelerometers into a single output. The models listed above are the ones used in our laboratory and can be substituted by other devices with the similar functional properties. A block diagram of the system is shown in the Figure 1. Three channels of the data acquisition and control interface are used for this procedure. The first channel is a digital–analog output channel (DAC output 0, Figure 1), which sends mono signal to the line input of the sound amplifier. Although the sound amplifier supports two stereo channels, each loudspeaker receives the same audio signal. The same channel also sends its output to an analog–digital input channel (e.g., ADC input 0, Figure 1) to record the output and to mark the precise moment of signal presentation for further analysis. Analog information about the animal’s movements from the four accelerometers is summed on the analog summing amplifier module, which further sends the signal to the third channel (e.g., ADC input 6, Figure 1) of the Power1401 interface.

**Figure 2:**
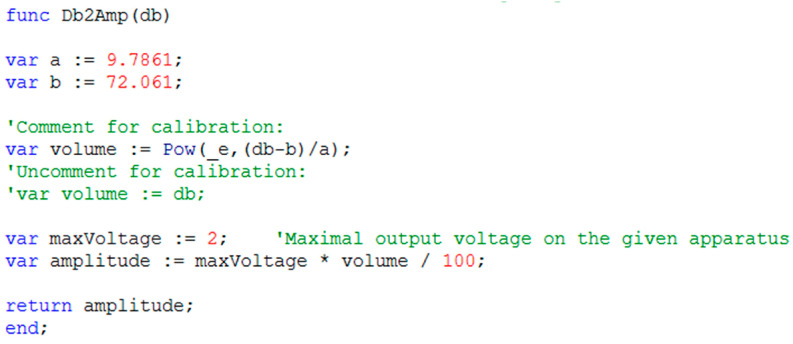
A fragment of the script’s code with the Db2Amp function needed for a sound level to voltage conversion and initial calibration.

**Figure 3:**
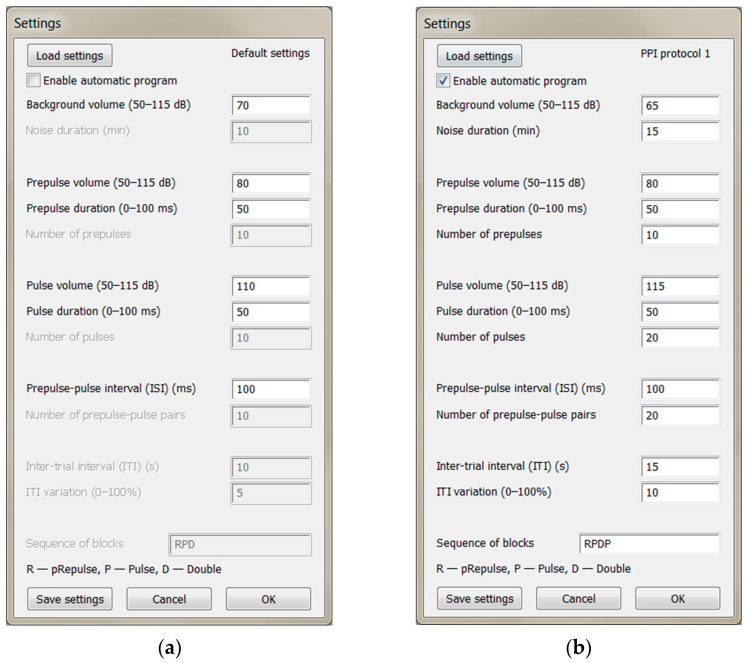
The script interface in the Spike2 program: (**a**) Manual mode; (**b**) Automatic mode.

Calibration of the sound volume is necessary to match specific hardware, including sound amplifier settings, loudspeakers, and cage acoustics. The Db2Vol function (Figure 2) converts custom sound volume settings in decibels to the linear range of 1–100. The function will further convert this value into the amplitude of the output voltage on the digital–analog channel. The variable “volume” is defined as percentage of the maximal output voltage on the given CED Power1401 apparatus that may be changed. During the calibration, measure the actual sound volume inside the cage using a sound level meter (DT-8820, CEM, Shenzhen, China). The calibration steps are as follows:

1. Assign “volume” directly to the “db” variable as shown in Figure 2;
2. In the Settings dialog box (Figure 3), set “Background volume” to 100, which sends the maximal possible voltage on the output channel, and run the script. Set the sound amplifier’s volume to the lowest level that yields the maximum sound volume necessary for the experiment (e.g., 120 dB) or possible, as measured with a sound level meter;
3. Set “Background volume” in the range of 1–10 with a step of 1 and in the range of 10–100 with a step of 5 or 10. For each value, measure the corresponding sound level in the cage. Plot the sound level as a function of the “Background volume” in the range of 1–100 and make a logarithmic approximation;
4. Assign the “a” and “b” variables in the “Db2Vol” function (Figure 2) to the corresponding values of the resulting equation of the logarithmic approximation:

db=a⋅ln(volume)+b

Throughout the experiment and between stimuli, white noise should be present in the background. Pulse signals also contain white noise with a higher amplitude. Signals can be presented either manually by pressing keys on the keyboard or automatically using a predefined program. In manual mode (Figure 3a), the experimenter can set the volume of the background noise, prepulse and pulse volume and duration, and the duration of the interstimulus interval (ISI) between the prepulse and pulse. The volume range indicated in the interface (Figure 3) is specific to laboratory conditions and is not strictly limited. The lower limit is determined by environmental noise and can be significantly reduced if an anechoic chamber is used. The upper limit depends on the maximum volume of the loudspeakers. If the volume of the prepulse is set below the level of the background noise, a gap-prepulse protocol can also be implemented [13]. To evoke each of the three types of signals, the corresponding keys on the keyboard are pressed: “r” for prepulse, “p” for pulse, and “d” for a double stimulus of prepulse and pulse. It is essential that the keyboard layout is set to English, and the current active window is the recording window in the Spike2 program. In addition to these settings, in automatic mode (Figure 3b), the experimenter can set the duration of habituation with background noise at the beginning of the experiment and the number of stimuli of each of the three types. The intertrial interval (ITI), which is the time between repetitive presentations of signals, together with its variation as a percentage of the initial ITI can also be customized.

The experimenter has the option to load and save specific protocol settings. The current protocol’s name is displayed in the upper-right corner of the Settings dialog. Additionally, a default settings file can be set by including its path at the start of the script.

The custom Spike2 script is published in open access and can be found on GitHub [16] and in the Supplementary Materials to the current article. It consists of a single PPI.s2s file and does not require any additional files. To add this script to the Spike2 software, it should be opened through the “Scripts” section in the interface.

### 2.2. An Example of Using the Script in Testing WT and DAT-KO Rats

**Figure 4:**
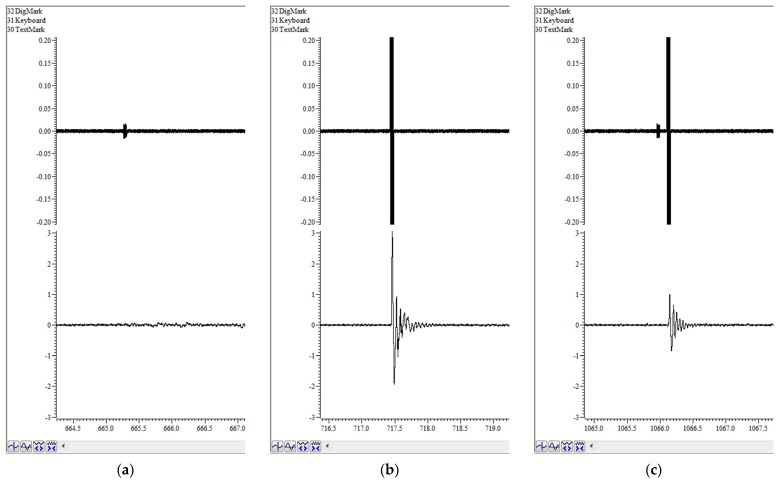
Screenshots of the Spike2 program, which show examples of ASR registration in response to different types of stimuli: (**a**) Single prepulse; (**b**) Single pulse; (**c**) Double prepulse+pulse. Upper channel—DAC output; lower channel—ADC input from vibration sensors with ASR.

Screenshots of the working window of the Spike2 program can be seen in Figure 4. The upper channel registers DAC output, which controls the volume of the loudspeakers. It shows the background white noise as well as pulses of different types, consisting of white noise of greater amplitude. The lower channel is the ADC input from vibration sensors on the platform with the animal. Between pulses, it records the animal’s background activity. There is no significant reaction to a single prepulse (Figure 4a), which means that the amplitude of the prepulse is appropriately chosen. Figure 4b shows a strong startle response to a single pulse. However, the response to a double pulse is apparently lower (Figure 4c) and reflects the PPI effect.

The ASR value was taken as the potential difference (in mV) between the minimum and maximum deviations observed no further than 200 ms from the moment of stimulus presentation. To calculate the PPI, the following formula was used:

$$\%\mathrm{PPI}=\left(1-\frac{\mathrm{prepulse}+\mathrm{pulse}\;\mathrm{response}\;\mathrm{amplitude}}{\mathrm{pulse}\;\mathrm{response}\;\mathrm{amplitude}}\right)\cdot 100\%.$$

As an example of the use of the script, we developed a series of experiments to assess attention impairment in dopamine knockout (DAT-KO) rats compared to wild-type (WT) rats was conducted.

We used 10 WT and 10 DAT-KO female rats of the same age (4 months). The protocol of the animal study was approved by the Ethics Committee for Animal Research of St Petersburg University, Saint Petersburg, Russia No. 131-03-10 of 22 November 2021. A day before the experiments, each rat was placed in the apparatus for 20 min and presented with 74 dB “white noise” for habituation. On the day of the experiment, each animal was presented with 74 dB “white noise” for 10 min, followed by ten acoustic stimuli of 78 dB volume and 50 ms duration (prepulse). The selected prepulse intensity did not cause any motor response in animals. Later, 20 acoustic stimuli with an intensity of 100 dB and a duration of 50 ms (pulse), causing a pronounced startle response, and 20 combinations of acoustic stimuli (prepulse+pulse) were presented. The decrease of the startle amplitude after a prepulse allowed calculation of prepulse inhibition index. The interval between prepulse and pulse stimuli was 100 ms. The interval between presentations of stimuli or paired stimuli varied between 10 to 14 s. Animal’s motor response was recorded from the platform by means of vibration sensors and synchronized with the presented signals using the Spike2 program with a CED Power1401 analog-to-digital converter (Cambridge Electronic Design).

## 3. Results

As an example, we measured the PPI in DAT-KO and DAT-WT female rats who lack a dopamine transporter and are characterized by a significant increase in the level of extracellular dopamine in the striatum [17]. The hyperactivity of these animals allows using them in model experiments for the ADHD syndrome investigations. During the experiments, we encountered difficulties in recording the reactions of DAT-KO rats, which are characterized by hyperactivity and an increased level of motor activity. This made it difficult to distinguish the motor reactions of animals from the background level of spontaneous movements. Mechanical restraint of DAT-KO rats causes significant stress, which can affect the results of the experiment. This problem was solved by installing a video camera synchronized with the recording of the animal’s motor reactions and the moments of presentation of sound signals, which made it possible to avoid the superposition of the recorded motor reactions on behavioral patterns of rats, such as rapid movements around the cage, grooming, and rearing.

**Figure 5:**
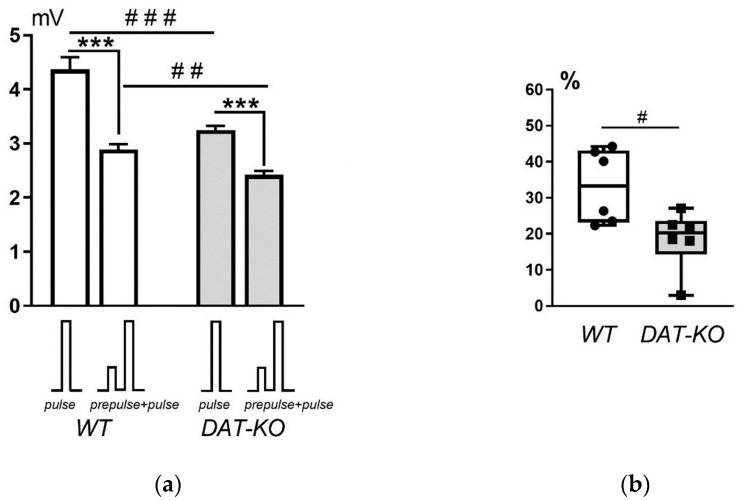
Comparison of acoustic startle reaction (ASR) and prepulse inhibition ratio (PPI) in WT and DAT-KO rats: (**a**) ASR amplitude (mV) on pulse and prepulse+pulse; (**b**) PPI (%), measured as the percentage of reduction in ASR amplitude on prepulse+pulse. # *p* < 0.05; ## *p* < 0.01; ***, ### *p* < 0.001; unpaired *t*-test.

The analysis of the results of testing DAT-KO and WT rats with the PPI script showed that the amplitude of the startle response (Figure 5a) in WT animals was significantly greater than that of DAT-KO rats in response to a single sound signal (pulse, 4.4 ± 0.2 mV vs. 3.2 ± 0.1 mV; *p* < 0.0001) and to the combination of two sound stimuli (prepulse+pulse, 2.9 ± 0.1 mV vs. 2.4 ± 0.1 mV; *p* = 0.0006). In both groups of animals (WT and DAT-KO), a significant decrease in startle amplitude was observed upon presentation of two sound stimuli compared to the response to a single stimulus (*p* < 0.0001).

Analysis of the magnitude of PPI showed that this parameter was significantly lower (*p* < 0.01) in knockout rats compared to the wild-type rats (Figure 5b). These observations indicate a reduced level of sensorimotor gating in DAT-KO rats, which are model animals for ADHD. Therefore, we can conclude that the PPI registration system developed in this study provides a reliable and valid method for assessing attention impairment in rodents.

## 4. Discussion

The presented software for CED interface offers an affordable and straightforward approach for performing experiments on PPI of acoustic startle response. Similar to earlier articles [9,10], this study showed high effectiveness and valid output results.

One of the primary advantages of the proposed software is its low cost for researchers who already have CED Power1401 and Spike2, thus making the method more accessible to scientists with limited budgets. Furthermore, the custom script can be modified and repurposed for various experimental designs and adapted to suit specific research needs. The custom script also offers flexibility in selecting the acoustic stimuli, volumes, and intervals between the stimuli, enabling researchers to perform more complex and detailed experiments.

Another significant advantage of the custom script is the possibility to perform simultaneous electrophysiological recordings during the PPI experiments. This advantage allows researchers to conduct electrophysiological experiments that require high temporal resolution, such as single-unit recordings, local field potentials (LFPs), and electroencephalogram (EEG) recordings, in conjunction with the PPI experiments. Such multimodal recordings enable the correlation of neural activity with behavioral responses, which can provide deeper insights into the underlying neural mechanisms of PPI.

Although the proposed solution requires experience with the Spike2 software and the CED Power1401 interface, it does not require a deep understanding of the Spike2 scripting language. A user-friendly graphical interface enables researchers to perform the experiments without extensive knowledge of the language and automates most of the experimental steps. The Spike2 software also has an in-built synchronization with video tracking, which improves the accuracy of the results and reduces the need for manual intervention.

Similar to commercial behavioral systems that support multiple recording with one control device and more than one soundproof chamber, our software can be up-scaled for simultaneous registration. DAC output controlled by the Spike2 script can be branched to different sound amplifiers, and information from accelerometers can be recorded with several ADC input channels. The inputs do not depend on the script and only require changing the sampling configuration.

However, the proposed solution has some limitations that need to be addressed. It relies on the quality and calibration of the existing equipment, including the sound equipment, which may affect the results accuracy and reproducibility. Additionally, while the presented custom script is flexible in adjusting various parameters, it lacks some features offered by commercial systems, such as the presentation of pure tones and automatic data analysis. Another benefit of commercial systems over our solution is that they offer customer support for configuration and installation, while we can only offer support in troubleshooting the program code.

Manual acquisition of the amplitudes of the startle responses may be significantly time-consuming and prone to errors. Moreover, in animals with increased motor activity, spontaneous movements may mask motor reactions in response to auditory stimuli. As a consequence, synchronous video recording of motor activity can be very useful in the control. Depending on the research task, animal holder can be used in order to reduce the background motor activity.

One potential improvement of the proposed software would be to develop an algorithm for automatic analysis of the results. Another possible improvement would be to incorporate new types of stimuli, in addition to white noise, and increase flexibility in the number of consecutive presentations. To minimize the impact of spontaneous movements and ensure accurate data acquisition, mechanical constraints should be used to restrict the animal’s locomotion without causing excessive stress.

We validated the proposed solution in an experiment with female DAT-KO rats. Most behavioral experiments are performed on males, but data obtained on females are also important for understanding the mechanisms of behavioral reactions and for adequate interpretation of drug testing results. According to the results of experiments in mice (males and females), DAT-KO animals demonstrated a reduced prepulse inhibition compared to WT mice [8,17]. According to the results of our previous experiments in male DAT knockout rats [9,10], ASR in response to single pulses was generally higher than to prepulse+pulse pairs, and PPI in male DAT-KO rats was significantly lower than in male WT rats. The data obtained in female WT and DAT-KO rats show the same tendency; ASR to single pulses is higher than ASR to prepulse+pulse pairs, and PPI in female DAT-KO rats is reduced compared to that in female WT.

In summary, the proposed software offers a cost-effective and flexible approach to performing PPI experiments with acoustic startle response. It has some limitations and requires at least Spike2 program, CED Power1401 or similar interface, and some additional laboratory equipment, but it also has a great potential for multimodal recording and complex experimental designs based on the Spike2 script. Future studies should aim at addressing the limitations of the proposed setup and at enhancing its capabilities, which would make the method more accessible and efficient for researchers.

## Data Availability

The raw data used in this study are available on request from the corresponding author.

## Supplementary Materials

The following supporting information can be downloaded at: https://www.mdpi.com/article/10.3390/mps6030057/s1, Script for acoustic startle response setup.
